
JOURNAL INFORMATION
==============================
NLM Title Abbreviation: Anim Front
Journal ID: Anim Front
NLM Journal ID: 101754918
Journal ID: anfron

Animal Frontiers: The Review Magazine of Animal Agriculture

ISSN: 2160-6056
EISSN: 2160-6064
Publisher: Oxford University Press

ARTICLE INFORMATION
==============================
PMCID: PMC12479243
DOI: 10.1093/af/vfaf012
Article ID: vfaf012
Article version: 1
Subjects: Society News, AcademicSubjects/SCI00960

News

Publication date: 2025 Jun
Electronic publication date: 2025 Apr 22
Volume: 15
Issue: 3
First page: 60
Last page: 65
Copyright: Copyright © 2025 American Society of Animal Science.
Copyright year: 2025
License: This is an Open Access article distributed under the terms of the Creative Commons Attribution License (https://creativecommons.org/licenses/by/4.0/), which permits unrestricted reuse, distribution, and reproduction in any medium, provided the original work is properly cited.
License URL: https://creativecommons.org/licenses/by/4.0/

Page count: 7

==============================

AMERICAN SOCIETY OF **ANIMAL SCIENCE**

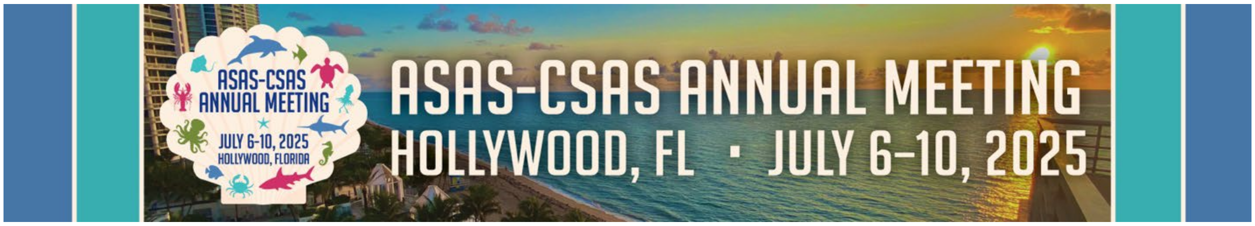

The 2025 Annual Meeting will take place at the Diplomat Beach Resort in Hollywood, Florida. When not in sessions, you are just steps away from soaking up the breezy South Florida lifestyle at the lagoon pool with ocean views of the alabaster beach.

The City of Hollywood is a beachfront community located in southeastern Broward County midway between Miami and Fort Lauderdale. Founded by Joseph Young in 1925, Hollywood is approximately 30 square miles in size and is Broward’s third-largest. Hollywood has an average annual high temperature of 83 degrees and low temperature of 68 degrees. Hollywood is home to over 60 parks, five golf courses, seven miles of pristine beaches, and the one-of-a-kind Hollywood Beach Broadwalk, a promenade stretching 2 miles along the Atlantic Ocean. Named one of America's Best Beach Boardwalks by Travel + Leisure magazine, this brick-paved thoroughfare hosts pedestrians, joggers, bicyclists, rollerbladers, and millions of others every year. Dozens of eateries and inns line the Broadwalk and the promenade also features the Hollywood Beach Theatre, a children’s water playground at Charnow Park, and many other attractions. Hollywood Beach also offers dozens of luxury hotels and condominiums such as the Diplomat Resort & Spa Hollywood.

Registration and housing information is available on the meeting website, https://www.asas.org/meetings/annual-2025.

[illegible]

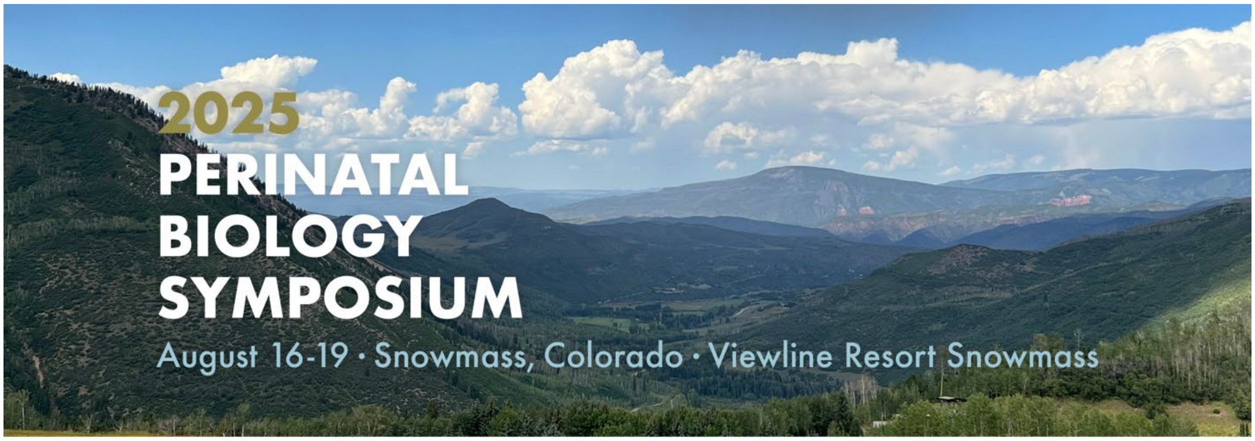

CHALLENGES AND SOLUTIONS FOR OPTIMIZING LONG-TERM HEALTH

Join us at the top of picturesque Snowmass Village, Colorado at the Viewline Resort Snowmass for the 2025 Perinatal Biology Symposium, August 16-19, 2025.

This Symposium will bring together clinicians, scientists and established senior and junior investigators from clinical medicine, basic research and applied livestock production, from around the world to report and discuss their findings in an atmosphere conducive to frank yet amicable exchange. Scientific exchange will occur across all levels via plenary sessions, poster sessions, trainee workshops and informal discussions, with time also dedicated to presentations by and recognition of trainees and early career investigators.
Abstract submission deadline is May 16, 2025.
More information can be found on the meeting website,
https://www.asas.org/meetings/perinatal-biology-symposium-2025

2026 World Congress on Genetics Applied to Livestock Production
July 12th-17th, 2026

The 2026 WCGALP will be held at the Frank Lloyd Wright designed Monona Terrace Community and Convention Center, overlooking the beautiful Lake Monona, just steps from the Wisconsin Capitol building in the heart of Madison, Wisconsin.

Stay up to date as new congress details come out by joining the meeting update list.

As a member of ASAS, we are sure you are aware of all the benefits that having a professional membership offer, including complimentary registration to monthly webinars and early access to the recordings, access to ASAS Journals, and discounted meeting registration to name a few. Remember to renew your ASAS membership or join ASAS to take advantage of these benefits.

More information on memberships can be found on the Membership website, https://www.asas.org/join-asas/individual-membership.

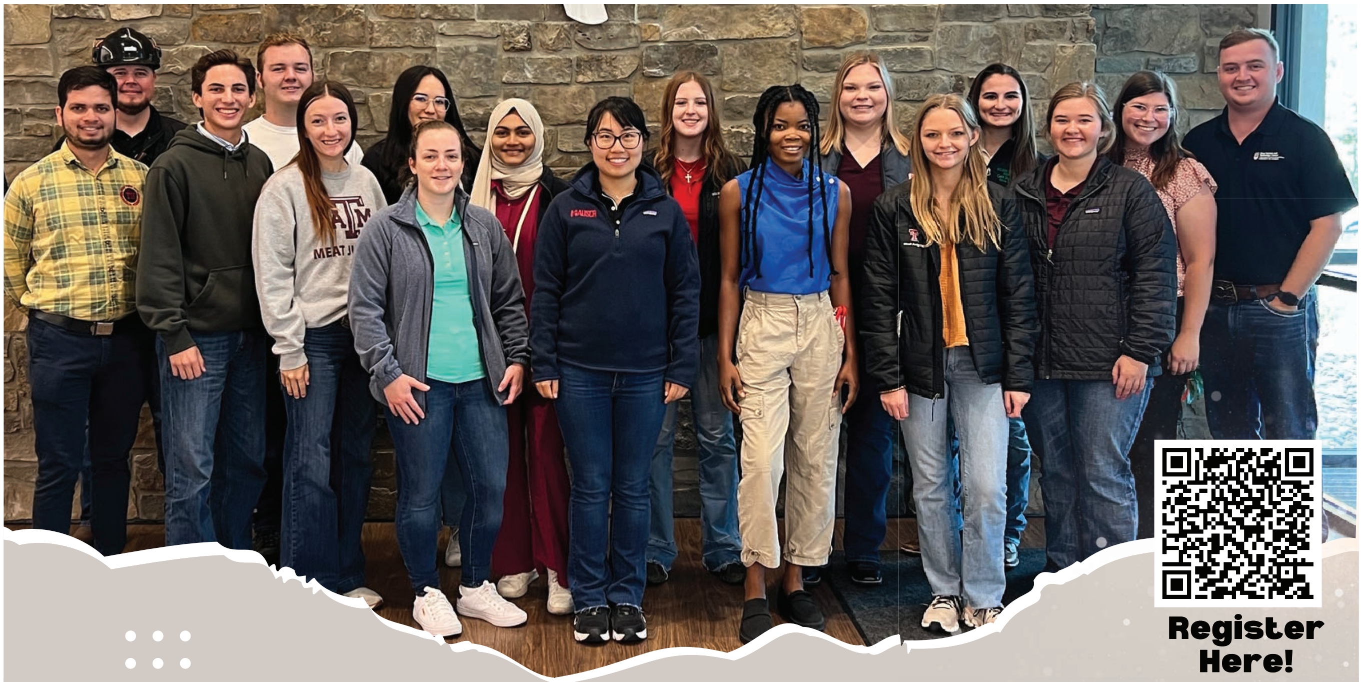

Register Here!

### National Student Leaders Connecting Conference

### REGISTER NOW

**JULY 23-25, 2025 | KANSAS CITY, MO**

Hosted with the 86th American Convention Of Meat Processors & Suppliers' Exhibition

AAMP AMERICAN ASSOCIATION OF MEAT PROCESSORS

STUDENT LEADERS SLC CONNECTING

American Meat Science Association

**PROGRAM SPONSORS**

AMERICAN MEAT SCIENCE ASSOCIATION DEVELOPMENT COUNCIL

Simmons

Nestlé PURINA

pork checkoff

UltraSource

Cargill

KEMIN Compelled by Curiosity™

For more information: information@meatscience.org or www.meatscience.org

EAAP
European Federation of Animal Science

**THE 76th EAAP ANNUAL MEETING WILL BE HELD IN INNSBRUK (AUSTRIA) FROM 25th TO 29th AUGUST 2025**

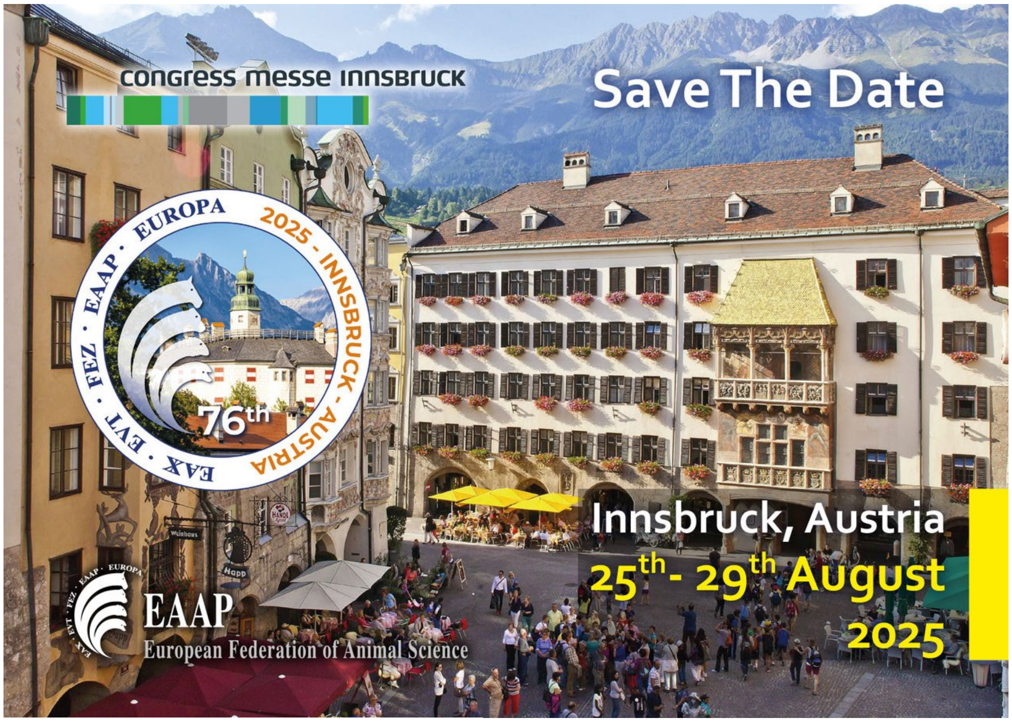

On behalf of the Austrian Organising Committee, we are very delighted to invite you to attend the 76th EAAP Annual Meeting 2024 to be held in Innsbruck (Austria), from 25th to 29th August 2025.
The sessions will be organized and jointly organized between the eleven EAAP Study Commissions to cover various areas of knowledge related to animal science, such as genetics, nutrition, physiology, animal health and welfare, precision livestock farming, horses, pigs, insects, livestock farming systems, cattle, sheep and goat. **Congress Website:** http:// www.eaap2025.org

The title of the Plenary session that will be organised on September 26th August is: "**Why Animal Farming Has Been Essential to Human Civilisation**". In this plenary session, we are thrilled to present a series of thought-provoking talks by distinguished invited speakers, each offering a unique perspective on the profound relationship between humans and animals throughout history. The following topics will be explored:

- **From Big Game to Animal Agriculture: How Humans Evolved to Need Animal Food**
  *Miki Ben-Dor* (Israel) will delve into the evolutionary journey that led humans to depend on animal-based food, examining how this shift influenced our development.
- **The Story of the Story of Domestication**
  *Greger Larson* (United Kingdom) will uncover the fascinating narrative of domestication, revealing how animals have shaped human societies across time.
- **How Animals Paved the Way for Modern Civilization** (draft title)
  *Haskel Greenfield* (Canada) will explore the crucial role animals have played in the advancement of civilization, providing insight into their impact on societal progress.

- **A Genomic History of Our Pets and Livestock: From Ancient Origins to Modern Breeding**
  *Laurent Frantz* (Germany) will offer a genomic perspective on the evolution of pets and livestock, tracing their origins and the development of modern breeding practices.

Join us for an enlightening session that takes a deep dive into the evolutionary and cultural significance of animals in human history.

Please follow us on Facebook and Twitter
https://www.facebook.com/EAAP.ORG
https://twitter.com/eaapofficial

INDIVIDUAL MEMBERSHIP OF EAAP

The EAAP membership is open to all scientists. It is a great opportunity to be update on the latest publications and other relevant information in the animal sector.
We have more than **6500 members** already!
Become a member of the most exciting international animal science network, joining us at
http://www.eaap.org/Content/Individual_Member_Information.html

THE EAAP NEWSLETTER FOR INDIVIDUAL MEMBERS IS NOW AVAILABLE ALSO IN CROATIAN, FRENCH, GERMAN, PORTUGUESE, ROMANIAN, SLOVENIAN, SPANISH, SLOVAK, ITALIAN AND POLISH!

WORLD ASSOCIATION for ANIMAL PRODUCTION

The World Federation of Animal Science Societies and Associations, **WAAP**, is an International Non-Governmental Organisation with special consultative status with FAO since 1974.
The WAAP was established in 1965 after the success of the 1st World Conference on Animal Production held in Rome in 1963 organised by EAAP under the patronage of FAO.
WAAP aims to encourage closer collaboration between the animal production organisations and to bring together scientists, educators, technicians and administrators with the objective of reviewing development concerns on the five continents.

The Lyon city at dusk

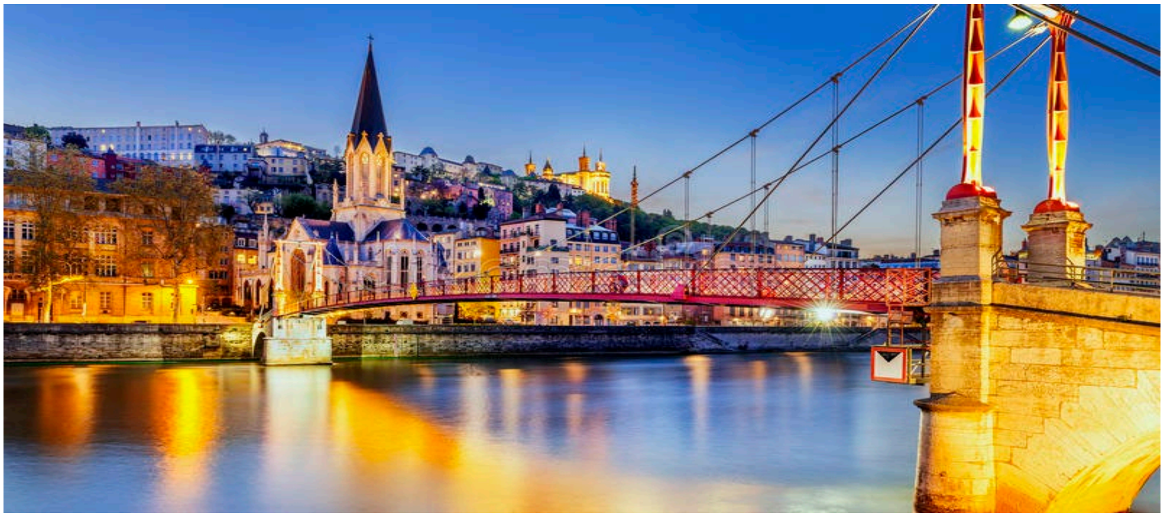

WAAP organises every five years a World Conference on Animal Production. Co-organized by EAAP and WAAP, the 2023 conference on “Climate change, biodiversity and global sustainability of animal production”, has been held in Lyon end August. It received 1900 abstracts among which 1200 were seleted for oral presentations in 96 sessions. More than 2200 delegates came to the conference. Ten sessions, organized or co-organized by WAAP, were video recorded and will be soon available by streaming on the EAAP Youtube channel. Two of them were especially attractive on the “Control of GHG emissions in ruminant farming” and on “Biodiversity as a lever for sustainable animal production”.

During the conference, WAAP delivered senior and junior awards and 3 student grants.

Philippe Chemineau ended his mandate after 5 years of presidency which was marked by the very positive entrance of WAAP in the Animal Frontiers consortium, but also negatively impacted by the COVID crisis.

Franck Dunshea was elected as the new President for 5 years. He will be in charge of organizing the next WCAP which will be held in Melbourne, Australia, in 2028.
